# Prospective longitudinal assessment of parotid gland function using dynamic quantitative pertechnate scintigraphy and estimation of dose–response relationship of parotid-sparing radiotherapy in head-neck cancers

**DOI:** 10.1186/s13014-015-0371-2

**Published:** 2015-03-15

**Authors:** Tejpal Gupta, Chandni Hotwani, Sadhana Kannan, Zubin Master, Venkatesh Rangarajan, Vedang Murthy, Ashwini Budrukkar, Sarbani Ghosh-Laskar, Jai Prakash Agarwal

**Affiliations:** Department of Radiation Oncology, Tata Memorial Hospital and ACTREC, Tata Memorial Centre, Parel, Mumbai, 400 012 India; Department of Epidemiology & Clinical Trials Unit - Clinical Research Secretariat, Tata Memorial Hospital and ACTREC, Tata Memorial Centre, Parel, Mumbai, 400 012 India; Department of Nuclear Medicine & Molecular Imaging, Tata Memorial Hospital and ACTREC, Tata Memorial Centre, Parel, Mumbai, 400 012 India

**Keywords:** Conformal radiotherapy, Dose–response, Salivary scintigraphy, Xerostomia

## Abstract

**Purpose:**

To estimate dose–response relationship using dynamic quantitative ^99m^Tc-pertechnate scintigraphy in head-neck cancer patients treated with parotid-sparing conformal radiotherapy.

**Methods:**

Dynamic quantitative pertechnate salivary scintigraphy was performed pre-treatment and subsequently periodically after definitive radiotherapy. Reduction in salivary function following radiotherapy was quantified by salivary excretion fraction (SEF) ratios. Dose–response curves were modeled using standardized methodology to calculate tolerance dose 50 (TD50) for parotid glands.

**Results:**

Salivary gland function was significantly affected by radiotherapy with maximal decrease in SEF ratios at 3-months, with moderate functional recovery over time. There was significant inverse correlation between SEF ratios and mean parotid doses at 3-months (r = −0.589, p < 0.001); 12-months (r = −0.554, p < 0.001); 24-months (r = −0.371, p = 0.002); and 36-months (r = −0.350, p = 0.005) respectively. Using a post-treatment SEF ratio <45% as the scintigraphic criteria to define severe salivary toxicity, the estimated TD50 value with its 95% confidence interval (95% CI) for the parotid gland was 35.1Gy (23.6-42.6Gy), 41.3Gy (34.6-48.8Gy), 55.9Gy (47.4-70.0Gy) and 64.3Gy (55.8-70.0Gy) at 3, 12, 24, and 36-months respectively.

**Conclusions:**

There is consistent decline in parotid function even after conformal radiotherapy with moderate recovery over time. Dynamic quantitative pertechnate scintigraphy is a simple, reproducible, and minimally invasive test of major salivary gland function.

**Electronic supplementary material:**

The online version of this article (doi:10.1186/s13014-015-0371-2) contains supplementary material, which is available to authorized users.

## Background

Definitive (chemo) radiotherapy is the contemporary standard of care in the non-surgical management of head and neck squamous cell carcinoma (HNSCC) [1,2]. The salivary glands are often incidentally irradiated during comprehensive irradiation of the head-neck cancers resulting in xerostomia that can adversely affect health-related quality-of-life (QOL) [3-5]. Xerostomia may result in poor oro-dental hygiene, altered taste sensation, and pain leading to difficulty in speaking, chewing and swallowing [6]. Xerostomia can be defined and graded [6,7] both subjectively according to patient’s symptoms (severity of dryness and/or response on stimulation) as well as objectively using quantified saliva production or excretion (salivary flow and/or scintigraphy). Stimulated salivary production is largely derived from the parotid glands while resting or unstimulated saliva is mostly produced by submandibular, sublingual, and various minor salivary glands [8]. Traditionally, salivary gland function has been assessed objectively by flow-rate measurements [9-11]. This can be performed at rest (unstimulated) or in response to administration of a sialogogue (post-stimulation). Collection of secretion from each parotid duct orifice via a cannula is the most common method of assessing individual parotid gland function. However, cannulation is an invasive procedure associated with a steep learning curve necessitating technical skill and expertise. It can at times be quite difficult and challenging [11], particularly in the post-treatment setting. Alternatively, whole mouth salivary function can be assessed by asking the patient to produce as much saliva as possible within a given period of time. Such measurementscan be uncertain and variable, with standard deviation of 20-30% reported for whole-mouth measurements [10]. In recent times, high-precision radiotherapy techniques such as three-dimensional conformal radiotherapy (3D-CRT) and intensity-modulated radiation therapy (IMRT) have gained immense popularity in HNSCC. IMRT produces highly conformal dose distributions with resultant substantial sparing of major salivary glands that can potentially reduce the incidence, duration, and severity of xerostomia with a positive impact on health-related QOL [12-15]. Parotid gland sparing can be further augmented using in-room image-guidance and periodic adaptive replanning during a course of fractionated radiotherapy [16]. With conformal techniques, individual salivary glands may be differentially spared, depending upon their proximity to high-risk areas. Thus, it is important to assess their functional status individually rather than as a whole as is typically assessed by whole-mouth measurements. Dynamic salivary gland scintigraphy using ^99m^Tc-pertechnate is a simple, reproducible, and minimally invasive test that provides quantitative estimates of parenchymal and excretory function of individual major salivary glands [17]. It can be a suitable alternative to salivary flow-rate measurements for quantification of post-radiotherapy salivary dysfunction.

## Aims

To report on prospective longitudinal assessment of functional changes in parotid glands using dynamic quantitative ^99m^Tc-pertechnate scintigraphy and estimate their dose–response relationship in a cohort of patients with HNSCC treated using parotid-sparing conformal radiotherapy techniques with or without platinum-based concurrent systemic chemotherapy.

## Materials and methods

Sixty previously untreated patients with early to moderately advanced squamous cell carcinoma of the oropharynx, larynx, or hypopharynx (stage T1-T3, N0-2b) were accrued and treated on an institutional review board approved prospective randomized controlled trial comparing 3D-CRT and IMRT. Suitable patients with loco-regionally advanced disease (bulky T2, T3, and/or node positive) also received concurrent chemotherapy. Cisplatin was administered once weekly as an intravenous infusion @30 mg/m^2^ with appropriate hydration, forced saline dieresis, and anti-emetic prophylaxis as per contemporary institutional standard of care. All patients provided written informed consent for participation in this mono-institutional randomized trial registered at Clinical Trials Registry-India (CTRI/2008/091/000045). Physician-rated acute salivary gland toxicity was the primary endpoint, while patterns of failure, loco-regional control, disease-free survival, overall survival, QOL, and late xerostomia were secondary endpoints. Details on target volume delineation, treatment planning, and delivery have been published previously [18,19]. Salivary gland toxicity (both acute and late xerostomia) was scored subjectively by the treating physicians using Radiation Therapy Oncology Group (RTOG) toxicity criteria [20].

### Salivary scintigraphy

**Figure 1:**
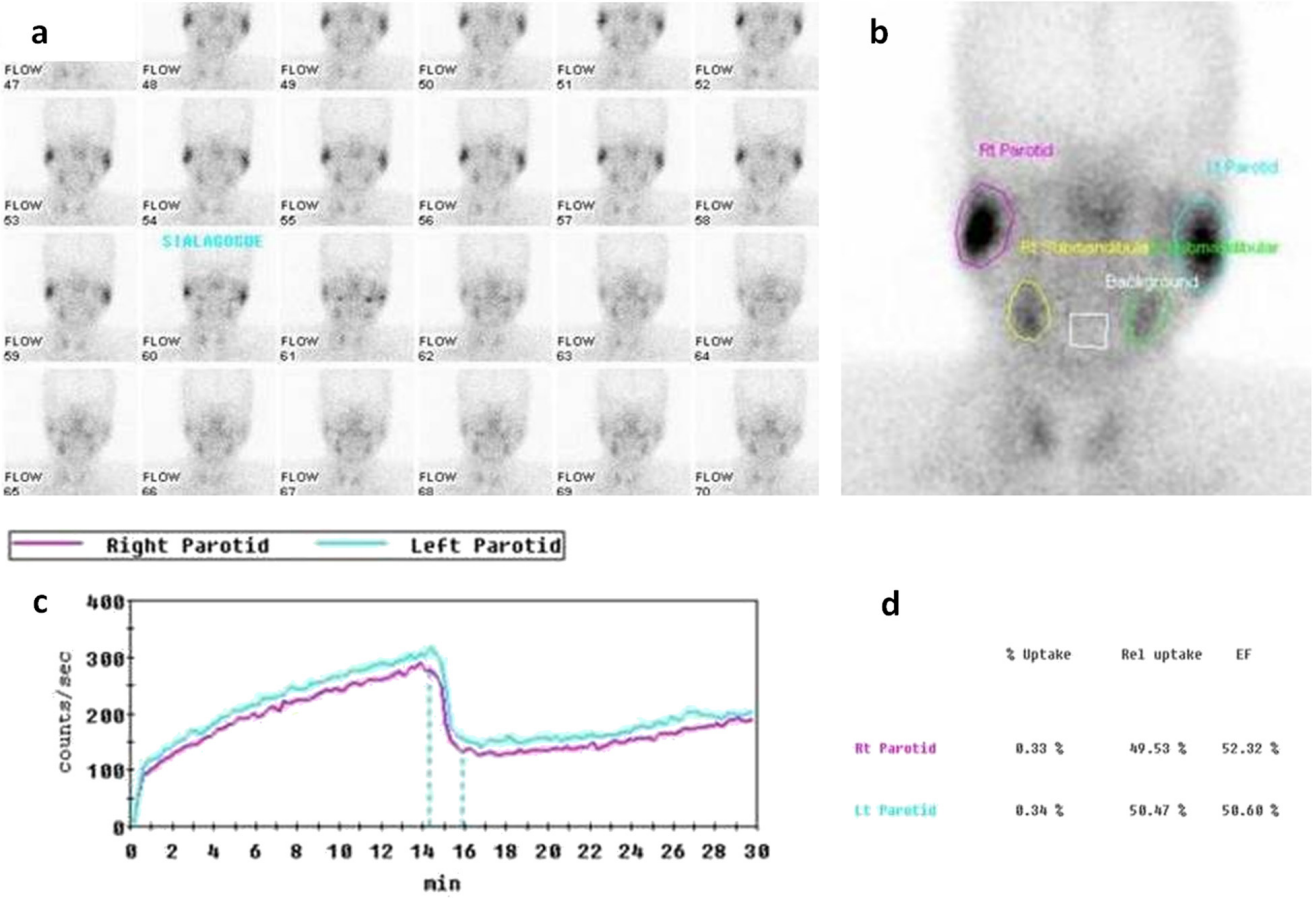
**Reframed dynamic images (1 minute per frame) showing increasing uptake in the salivary glands and subsequent washout after sialogogue administration halfway through the study. (a)**: Regions of interest drawn around major salivary glands **(b)** with background correction performed using the midline neck region. Typical pre-treatment time-acitivty curves **(c)** for right and left parotid glands. Note the steady and progressive increase in uptake immediately following injection of ^99m^Tc-pertechnate. Stimulation of salivary secretion by sialogogue (15-minutes post-injection) leads to decline in detectable counts within the glands. Percentage uptake and relative uptake of individual parotid gland **(d)**. Salivary excretion fraction (SEF) is quantified by calculating maximal excretory activity per gland as a fraction of its maximal uptake **(d)**.

All patients underwent salivary gland scintigraphy using ^99m^Tc-pertechnate prior to initiation of definitive (chemo)radiotherapy. During the initial part of the study, scintigraphy was done using a semi-quantitative methodology, precluding accurate quantification of salivary function. Subsequently, dynamic quantitative ^99m^Tc-pertechnate scintigraphy was performed according to the method described by Klutman [17] in the remaining 41 patients (82 parotid glands) that constitute the dataset of this analysis. Quantitative assessment of the salivary function was performed at baseline (pre-treatment) and subsequently longitudinally at pre-specified time-points on follow-up viz. 3-months (n = 41); 12-months (n = 38); 24-months (n = 35); and 36-months (n = 32) after completion of definitive (chemo)radiotherapy. Scintigraphy studies were performed with the patient in the supine position under a gamma-camera (Infinia Hawkeye, GE Healthcare, Waukesha, USA) with low-energy high-resolution collimators. No oral stimulus was permitted for 60 minutes before imaging. After intravenous administration of 15 mCi (200 MBq) ^99m^Tc-pertechnetate, 30-second sequential frames (anterior view) were acquired and stored in the computer system. Fifteen minutes after injection, salivary stimulation was provided by ingestion of 5 ml of sialogogue (lemon juice). The study was continued for another 10 minutes after sialogogue administration. For analysis of the data, regions of interest were drawn around the right and left parotid and submandibular glands by nuclear medicine physicians and corresponding time-activity curves generated. Background correction was performed using the midline neck region. Time-activity curves were fitted to exponential functions. Salivary excretion fraction (SEF) of an individual salivary gland was quantified by calculating the maximal excretory activity per gland as a fraction of maximal uptake (Figure 1).

### Dose–response analysis

For the dose–response analysis, it was assumed that the glands within an individual patient would not influence each other. Reduction in salivary gland function after (chemo)radiotherapy was described by the relative SEF or SEF ratio defined as the ratio of SEF at time-point‘t’ after treatment compared to the baseline SEF (pre-treatment) x 100%. SEF ratios at different time-points on follow-up (3, 12, 24, and 36-months) were correlated with mean parotid doses. An SEF ratio <45% [21] was used as an objective scintigraphic criteria to define severe salivary toxicity. This has been shown to correlate best with salivary flow-rate measurements [21] wherein flow-reduction to <25% of pre-treatment output is regarded as severe salivary gland toxicity. Dose–response analysis was restricted to individual parotid glands in this study. Submandibular glands were not considered for such analyses as they had neither been contoured nor given any dose-volume constraints during radiotherapy planning and optimization. Data were fitted to the Lyman-Kutcher-Burman (LKB) model [22,23] for calculating normal tissue complication probability (NTCP). Briefly, this model assumes that the probability of complications after uniform irradiation of a specified partial volume of an organ follows a sigmoid dose–response relationship. Three parameters in this model are ‘n’, ‘m’, and tolerance dose 50 (TD50). The parameter ‘n’ accounts for the volume effect of an organ and was considered as 1 for the purpose of this analysis assuming parallel architecture of the parotid glands. The parameter ‘m’ describes the slope of the dose–response curve. The TD50 of partial volume ‘v’ is the dose resulting in 50% probability of a complication for uniform irradiation of that partial volume ‘v’. The model requires input of a single parotid gland dose. The multi-step dose-volume histogram (DVH) was transformed to a single-step DVH with an effective partial volume irradiated uniformly by a reference dose. The inputs to the model were transformed DVH and parotid gland function that was adjusted as a binary response variable on the basis of each individual gland. A maximum likelihood method was used to fit the model to the complication data and find the best estimate and 95% confidence intervals (95% CI) for the model parameters. In an exploratory analyses, dose–response curves were also generated to estimate TD50 values and the corresponding slope (m) using different SEF ratios to define severe salivary gland toxicity (ranging from SEF ratio <75% to <25%). Agreement between subjective xerostomia scores (RTOG grading) and objective scintigraphic criteria (SEF ratio <45%) was tested using the kappa statistic.

## Results

Relevant demographic, clinical, and dosimetric characteristics of the study cohort (n = 41) are described in Table 1. The mean and standard deviation (SD) of mean doses to ipsilateral and contralateral parotid glands were 48.3Gy (13.0) and 39.7Gy (12.8) respectively. With IMRT, the median and its inter-quartile range (IQR) of mean doses to the ipsilateral parotid gland was 37.2Gy (30.4-49.0Gy) compared to 59.3Gy (51.2-63.8Gy) with 3D-CRT (p < 0.001). The contralateral parotid gland was also spared significantly with IMRT. The median of mean doses with its IQR to the contralateral parotid gland was 28.1Gy (25.2-30.4Gy) with IMRT which was significantly lesser than 53.3Gy (43.8-56.4Gy) with 3D-CRT (p < 0.0001).

**Table 1:** **Demographic and treatment characteristics of study cohort (N = 41)**

***Characteristics***	***Number***
***Age:***	
Median	54 years
Range	33-65 years
***Gender:***	
Male	37 (90.2%)
Female	04 (09.8%)
***Primary site:***	
Oropharynx	23 (56.1%)
Larynx	10 (24.4%)
Hypopharynx	08 (19.5%)
***Laterality (epicentre):***	
Right	22 (53.7%)
Left	18 (43.9%)
Midline	01 (02.4%)
***American Joint Committee on Cancer (AJCC) staging:***	
Stage II	09 (21.9%)
Stage III	17 (41.5%)
Stage IV	15 (36.6%)
***Radiotherapy technique:***	
Three-Dimensional Conformal Radiotherapy (3D-CRT)	20 (48.8%)
Intensity-Modulated Radiation Therapy (IMRT)	21 (51.2%)
***Median (inter-quartile range) of mean parotid dose:***	
Ipsilateral parotid	50.0 Gy (36.2-59.7)
Contralateral parotid	35.4 Gy (28.0-53.5)
***Concurrent chemotherapy:***	
Yes	38 (92.6%)
No	03 (07.4%)

**Figure 2:**
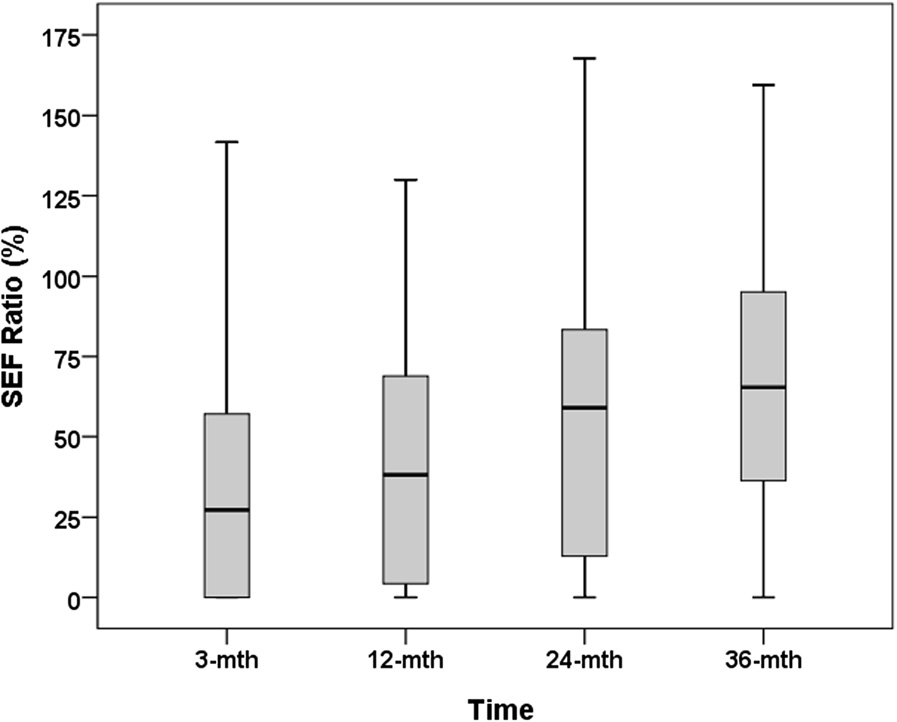
**Boxplot showing median salivary excretion fraction (SEF) ratios at 3-months, 12-months, 24-months, and 36-months after (chemo) radiotherapy.** Note the moderate recovery of salivary function continuing till 2-years post-treatment.

The pre-treatment (baseline) SEF was normally distributed for both parotid glands with a mean value of 50.1% (SD = 14.1). However, considerable variability of parotid gland output was noted with baseline SEFs ranging from 10-70%. No significant pre-treatment differences were found between the right and left parotid gland SEFs. Baseline scintigraphic parameters were not dependent on age, gender, or stage. The parenchymal as well as the excretory function of all major salivary glands was significantly affected by (chemo)radiotherapy with resultant decrease in SEF ratios at 3-months following completion of therapy. At 12-months post-treatment, there was modest functional recovery of the parotid glands (contralateral > > ipsilateral), which improved progressively further till 24-months, but reached a plateau somewhat thereafter. The median SEF ratios (IQR) of the parotid glands were 25.7% (0–55.8%), 38.2% (3.8-68.9%), 59.0% (8.4-83.6%), and 65.3% (29.4-95.4%) at 3-months, 12-months, 24-months and 36-months respectively (Figure 2) after (chemo)radiotherapy indicating substantial recovery of salivary function over time, mostly within the first two years on follow-up.

**Figure 3:**
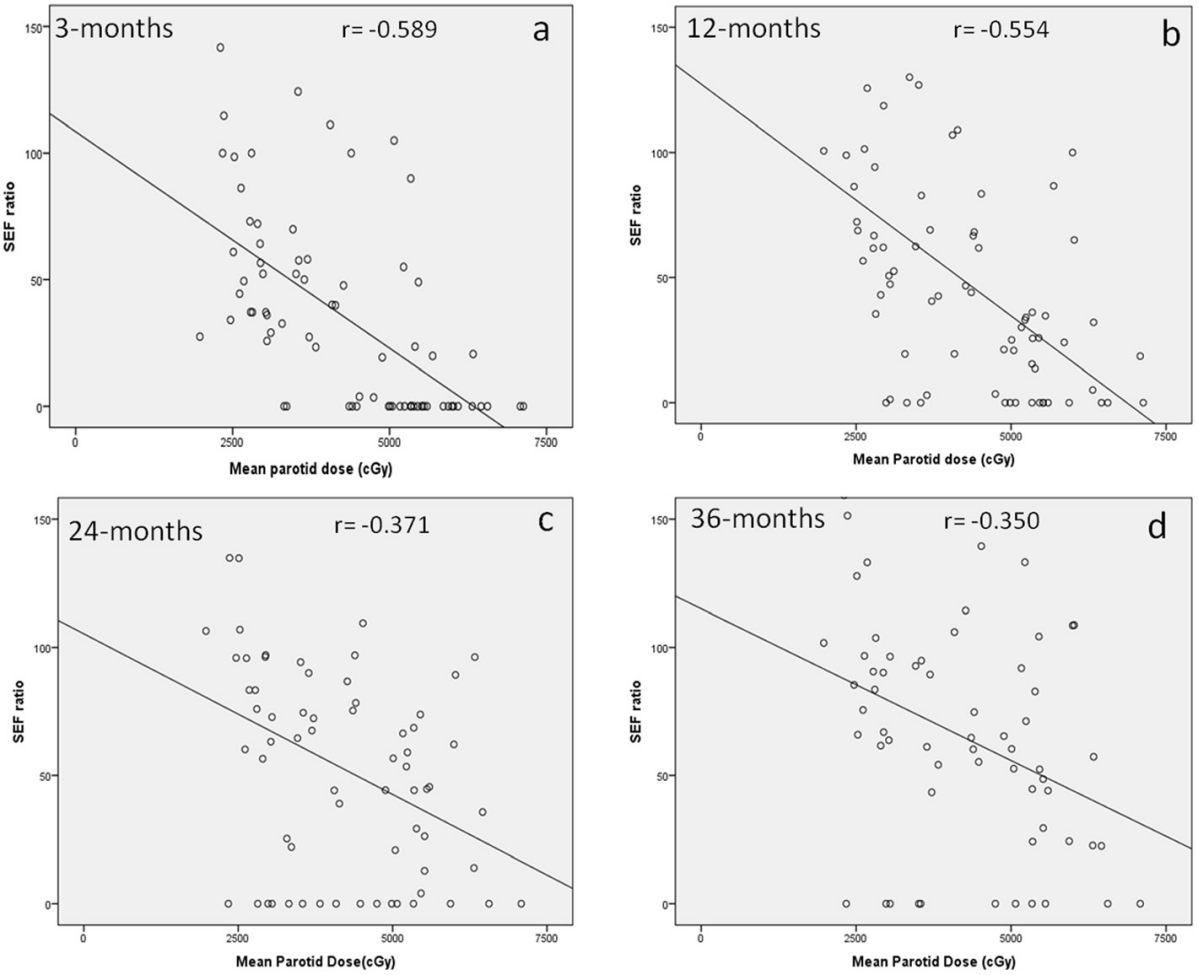
**Salivary excretion fraction (SEF) ratio as a function of mean parotid dose at 3-months (a), 12-months (b), 24-months (c), and 36-months (d) respectively.** Note the significant inverse correlation between the two at all time-points.

**Figure 4:**
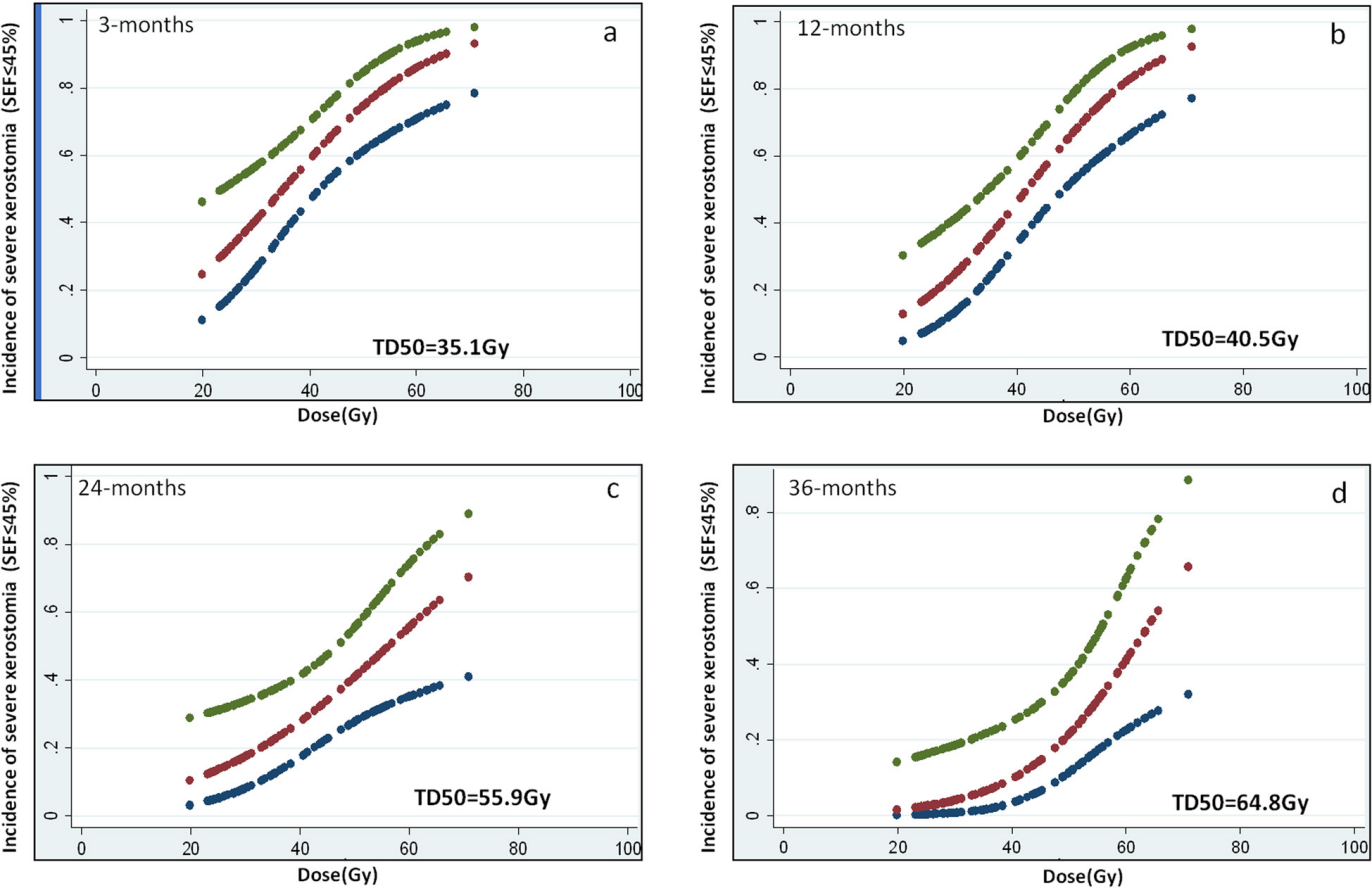
**Fitted dose–response curves for normal tissue complication probability of severe xerostomia (defined as SEF ratio <45%) as a function of mean parotid dose at 3-months (a), 12-months (b), 24-months (c), and 36-months (d) respectively.** Upper and lower curves represent 95% confidence intervals for the fitted model.

There was significant inverse correlation (Figures 3a-d) between SEF ratios and mean parotid doses at 3-months (r = −0.589, p < 0.001); 12-months (r = −0.554, p < 0.001); 24-months (r = −0.371, p = 0.002); and 36-months (r = −0.350, p = 0.005) respectively (see online Additional file 1: Table S1). Using a post-treatment SEF ratio <45% as the scintigraphic criteria to define severe salivary gland toxicity [21], the estimated TD50 (95% CI) values for the parotid glands at 3-months, 12-months, 24-months, and 36-months were 35.1Gy (23.6-42.6Gy), 41.3Gy (34.6-48.8Gy), 55.9Gy (47.4-70.0Gy) and 64.3Gy (55.8-70.0Gy) respectively (Figure 4a-d). The upper limits of the 95% CI of TD50 estimates at 24 and 36 months could not be computed accurately, as the dose–response curves lost some of their sigmoidal nature and became somewhat flatter over time. The corresponding ‘m values (slope of the dose–response curve) were 0.48, 0.38, 0.44, 0.38 for the four time-points respectively. Using the Quantitative Analysis of Normal Tissue Effects in the Clinic (QUANTEC) 20/20 rule [7], the incidence of severe toxicity (SEF < 45%) was estimated at 23% at 3-months, but decreased to 13% and 9% at 12-months and 24-months respectively, providing external validation of the QUANTEC guidelines in predicting a low probability of severe xerostomia. There was poor to weak agreement between subjective scores (physician-rated RTOG salivary gland toxicity) and objective scintigraphic criteria (toxicity defined as SEF ratio <45%) at all four post-treatment time-points (see online Additional file 2: Table S2). Results of the exploratory analyses estimating the TD50 values and the slope (m) of the dose–response curve at all four time-points using different SEF ratios to define severe salivary gland toxicity are also summarized (see online Additional file 3: Table S3).

## Discussion

Curative-intent radiotherapy for head-neck cancers often leads to irreversible impairment of salivary function and consequent xerostomia that adversely affects health-related QOL [3-5]. This decline in salivary function occurs even after parotid-sparing conformal radiotherapy albeit to a lesser degree (both in terms of incidence and severity) particularly with IMRT [19] with substantial recovery over time. The use of IMRT in clinical practice has resulted in improved tolerance to treatment [24] for patients with head-neck cancer and reduced delayed or late effects. Dose–response relationship for major salivary glands has traditionally been based on salivary flow-rate measurements [9-11]. In particular, a strong correlation has been shown between the mean parotid dose and residual post-radiotherapy salivary function [7]. There is a gradual decrease in salivary flow with increasing mean parotid dose. Minimal functional impairment occurs at mean doses <10-15 Gy, increasing doses (in the range of 20-30Gy) leads to progressive deterioration with severe xerostomia occurring at mean parotid doses of >40Gy. The TD50 for the endpoint of severe xerostomia (traditionally defined as reduction in salivary flow rate to <25% of pre-treatment value) has been quite variable with estimates ranging from 20–45 Gy [7].

Eisbruch et al. [9] described a steep dose–response relationship for the parotid glands in a cohort of 88 patients treated with IMRT with estimated 1-year TD50 of 28Gy using salivary flow-rate measurements. Using similar methodology, Chao et al. [10] also reported a TD50 of 32Gy. In contrast, Roesink et al. [11] found no threshold dose in 108 patients of head-neck cancer treated with conventional techniques but reported a TD50 of 39Gy at 1-year using flow-data. The largest dataset of parotid gland function measurements at 1-year (combining the Michigan and Utrecht experience) reported a TD50 of 39.9 Gy and a complication probability of 17-26% with mean parotid doses in the range of 25–30 Gy [25].

While flow-rate measurements have remained the benchmark for assessment of salivary function, given their limitations, dynamic quantitative pertechnate scintigraphy has emerged as a simple, reproducible, and minimally invasive test for quantification of post-radiotherapy salivary function of individual major salivary glands. Unlike, salivary flow-rate measurements, there has been a lack of consensus on the definition of severe salivary toxicity using scintigraphic criteria. In the largest scintigraphic dataset (n = 96), Roesink et al. [21] reported significant correlation between SEF ratios and mean parotid doses, both in early (6-weeks) and later (1-year) follow-up. They also modeled the dose–response curves using different SEF ratios to define severe salivary toxicity due to relative lack of previous reports fitting scintigraphic data to NTCP models. Severe salivary toxicity defined as SEF ratio <45% gave TD50 estimates that were comparable to their flow-data at 6-weeks and 1-year after treatment. The Heidelberg group has consistently used SEF ratio <50% to define severe salivary toxicity and TD50 (95% CI) estimates for the parotid gland of about 35Gy (95% CI = 20-45Gy) between 2–6 months post-treatment [26-28]. Recent times have witnessed more widespread use of salivary scintigraphy for post-radiotherapy assessment of salivary dysfunction. The estimated tolerance doses for the parotid gland in selected studies [21,26-31] using quantitative salivary scintigraphy are summarized in Table 2. The reported variability in scintigraphy-based TD50 values is somewhat lesser compared to flow-based estimates. Also scintigraphy-based TD50 estimates have generally tended to be higher than their flow-based counterparts. At this point, salivary scintigraphy cannot be considered superior to salivary flow-rate measurements, but can be a viable practical alternative. The reported variation in reported TD50 values (both for salivary flow data as well as scintigraphic data) could be a result of differences in radiotherapy techniques and resultant dose distributions, fraction-size effects, intra-gland sensitivity, use of concurrent chemotherapy, methods of measurement, definition of toxicity, time-points of assessment, and NTCP models used for such calculation. Semenenko and Li [32] in a pooled analysis of published clinical data to provide population LKB-NTCP model parameters for incorporation in treatment planning estimated a TD50 (95% CI) of 31.4Gy (29.1-34.0Gy) for the endpoint of reduction in stimulated salivary flow below 25% within six months after radiotherapy.

**Table 2:** **Studies estimating tolerance dose 50 (TD50) of parotid glands using salivary scintigraphy**

***Study (ref)***	***Number of patients (N)***	***Mean parotid dose***	***Salivary scintigraphy criteria for defining severe xerostomia***	***Tolerance Dose 50 (95% CI)***	
				***6 weeks-6 months***	***1-year***
#Roesink [21]	96 (conv)	33.14Gy	SEF ratio <45%	29Gy (25-34Gy)	43Gy (37-51Gy)
Munter [26]	18 (IMRT)	NR	SEF ratio <50%	34.8Gy (27.6-42Gy)	NR
Munter [27]	33 (conv)	60.6Gy	SEF ratio <50%	36.4Gy (20.5-42.3Gy)	NR
	19 (IMRT)	27.7Gy	SEF ratio <50%	35Gy (28-42Gy)	
*Rudat [28]	34 (conv)	60.7Gy	SEF ratio <50%	NR	51.1Gy (43.5-58.7Gy)
	31 (IMRT)	30.9Gy			
Tenhunen [29]	20 (IMRT)	27.6Gy	SEF ratio <50%	40.3Gy (30–53.6Gy)	39.2Gy (27.9-50.2Gy)
Kapanen [30]	25 (IMRT)	23.2Gy	SEF ratio <50%	30.4Gy (23.2-37.6Gy)	NR
Chen [31]	31 (IMRT)	51.7Gy IL	SEF ratio <45%	NR	43.6Gy (41.3-45.9Gy)
		36.7Gy CL			
Present study	41 (3D-CRT and IMRT)	48.3Gy IL	SEF ratio <45%	35.1Gy (23.6-42.6Gy)	41.3Gy (34.6-48.8)
		39.7Gy CL			

CI = confidence interval; SEF = salivary excretion fraction; conv = conventional; 3D-CRT = three dimensional conformal radiotherapy; IMRT = intensity modulated radiation therapy; NR = not reported; IL = ipsilateral; CL = contralateral.

#First report correlating salivary flow measurements with scintigraphic dataset; SEF ratio <45% best correlated with flow data becoming the benchmark scintigraphic criteria defining severe xerostomia.

*Updated results from previous publication (ref) reporting delayed xerostomia; conventional radiotherapy plus amifostine group has been excluded from these estimates.

## Strengths and limitations

The TD50 (95% CI) estimates for the parotid glands in this study were derived from a prospective cohort of patients. Hence they do not suffer from inherent limitations of any retrospective analyses. The relatively wide dispersion of mean parotid doses in the study allowed for more robust curve fitting at both ends of the spectrum. Serial follow-up provided an opportunity to estimate longitudinal recovery of salivary function over time and calculate TD50 values at longer follow-up times (2 and 3-years) than is generally reported in the literature (typically up to 1-year). However, the dose–response curves became somewhat flatter over time precluding accurate computation of the upper limits of the 95% CIs of the TD50 estimates at 24 and 36-months. Dose–response analyses was restricted to parotid glands only in the study thereby precluding such modeling for submandibular salivary glands which are the major contributors to salivation in the resting state. Intentional sparing of contralateral submandibular salivary gland leads to better preservation of salivary function without any increased risk of marginal failure in the vicinity of the spared gland [33]. Vast majority of patients in the study also received concurrent weekly cisplatin that could possibly influence salivary toxicity. Although cisplatin alone *per se* does not cause significant salivary dysfunction, its use as a sensitizer concurrently with radiotherapy increases biologically delivered doses potentially enhancing radiotherapy-induced salivary gland toxicity. In addition to physician-rated xerostomia, salivary scintigraphy was used as an objective test to quantify post-radiotherapy salivary dysfunction. However, this study did not use salivary-flow measurements generally considered the benchmark for such quantification. Lack of consensus criteria for defining severe salivary toxicity using scintigraphy was another limitation of the study. Nevertheless, various SEF ratio cut-offs were used to define salivary toxicity in an exploratory analyses, although SEF <45% was used in the final analysis, interpretation, and reporting. What is also reassuring is that the TD50 estimates (particularly at 1-year) obtained in this study are pretty similar to previously published data of salivary scintigraphy. Although, patients filled QOL forms at baseline and serially longitudinally on follow-up, a xerostomia-specific questionnaire was not used in this study to assess patient-reported outcomes (self-rated xerostomia). Finally, there was poor to weak agreement between subjective xerostomia scores and objective scintigraphic criteria suggesting that observer-based monitoring may underestimate actual xerostomia mandating the need for patient-reported outcomes for such estimation. Large variability in salivary gland function between patients, poor correlation between objective and subjective assessment of salivary toxicity, and limitations of statistical modeling make accurate prediction of salivary dysfunction in an individual patient difficult and challenging.

The tolerance dose estimates for different measures used to describe high-grade xerostomia viz. salivary flow-rates, observer-rated subjective xerostomia, and patient-reported QOL outcomes on a xerostomia-specific questionnaire can be very different. Miah and colleagues [34] reported increasing TD50 values from parotid flow-rates (23.4Gy), subjective xerostomia (33.3Gy), RTOG-graded subjective xerostomia (42.9Gy), and patient-reported QOL outcomes (51.6Gy). In the largest analysis (n = 237 patients) using patient-reported QOL data of moderate to severe xerostomia [35], the fitted dose–response curves (LKB-NTCP model) yielded a TD50 of 37.8Gy and 43.9Gy at 3-months and 1-year respectively. Reassuringly, another study [36] that used patient-reported QOL outcomes for fitting to the dose–response curve for NTCP of incidence of ≥ grade 3 xerostomia reported a TD50 of 44.1Gy for the parotid glands 1-year after radiotherapy which was very similar to their TD50 value of 43.6Gy [31,36] estimated using salivary scintigraphy (SEF ratio <45%).

## Conclusions

Xerostomia remains an important toxicity following curative-intent irradiation of head-neck cancers. There is consistent and significant decline in parotid gland function even after conformal radiotherapy, albeit to a lesser degree, particularly with IMRT, compared to conventional radiotherapy. However, parotid gland function recovers moderately on longer follow-up, as evidenced by progressively higher SEF ratios and TD50 values over time. Dynamic ^99m^Tc-pertechnate scintigraphy is a simple, reproducible, and minimally invasive test of major salivary gland function that may be a suitable alternative to salivary flow-rate measurements in clinical practice for quantification of post-radiotherapy salivary dysfunction.

## Additional files

Additional file 1:
**Correlation of SEF ratio with mean parotid dose at various time-points on follow-up.**
Additional file 2:
**Test of agreement between subjective xerostomia scores and objective scintigraphic criteria.**
Additional file 3:
**Estimated tolerance dose 50 (TD50) in Gy and corresponding slope (m) of the dose–response curve for the parotid gland at different time-points on serial follow-up using different SEF ratios to define severe salivary toxicity.**
