# Supplementary material for: Prospective longitudinal assessment of parotid gland function using dynamic quantitative pertechnate scintigraphy and estimation of dose–response relationship of parotid-sparing radiotherapy in head-neck cancers
Source: Radiat Oncol. 2015 Mar 15;10:67. doi: 10.1186/s13014-015-0371-2 (PMC4373026; doi:10.1186/s13014-015-0371-2)
Supplement: Additional file 1: — Correlation of SEF ratio with mean parotid dose at various time-points on follow-up. [file 13014_2015_371_MOESM1_ESM.docx]

**Additional file 1: Table S1: Correlation of SEF ratio with mean parotid dose at various time-points on follow-up**

| ***SEF ratio*** | ***Mean Parotid dose*** | |
| --- | --- | --- |
|  | ***Pearson’s correlation coefficient***  ***(95% confidence interval)*** | ***p-value*** |
| 3-month follow-up | -0.589 (-0.716 to -0.423) | <0.001 |
| 12-month follow-up | -0.554 (-0.692 to -0.376) | <0.001 |
| 24-month follow-up | -0.371 (-0.558 to -0.148) | 0.002 |
| 36-month follow-up | -0.350 (-0.550 to -0.112) | 0.005 |
