# Supplementary material for: Prospective longitudinal assessment of parotid gland function using dynamic quantitative pertechnate scintigraphy and estimation of dose–response relationship of parotid-sparing radiotherapy in head-neck cancers
Source: Radiat Oncol. 2015 Mar 15;10:67. doi: 10.1186/s13014-015-0371-2 (PMC4373026; doi:10.1186/s13014-015-0371-2)
Supplement: Additional file 2: — Test of agreement between subjective xerostomia scores and objective scintigraphic criteria. [file 13014_2015_371_MOESM2_ESM.docx]

**Additional file 2: Table S2: Test of agreement between subjective xerostomia scores and objective scintigraphic criteria**

| ***Time-point*** | ***kappa value (95%CI)*** | ***p-value*** | ***Interpretation*** |
| --- | --- | --- | --- |
| 3-months | 0.387 (0.087-0.687) | 0.008 | Weak agreement |
| 12-months | 0.377 (0.073-0.682) | 0.011 | Weak agreement |
| 24-months | 0.266 (-0.080-0.613) | 0.073 | Poor agreement |
| 36-months | 0.310 (-0.068-0.689) | 0.057 | Poor agreement |
