# Supplementary material for: Prospective longitudinal assessment of parotid gland function using dynamic quantitative pertechnate scintigraphy and estimation of dose–response relationship of parotid-sparing radiotherapy in head-neck cancers
Source: Radiat Oncol. 2015 Mar 15;10:67. doi: 10.1186/s13014-015-0371-2 (PMC4373026; doi:10.1186/s13014-015-0371-2)
Supplement: Additional file 3: — Estimated tolerance dose 50 (TD50) in Gy and corresponding slope (m) of the dose–response curve for the parotid gland at different time-points on serial follow-up using different SEF ratios to define severe salivary toxicity. [file 13014_2015_371_MOESM3_ESM.docx]

**Additional file 3: Table S3: Estimated tolerance dose 50 (TD50) in Gy and corresponding slope (m) of the dose-response curve for the parotid gland at different time-points on serial follow-up using different SEF ratios to define severe salivary toxicity**

| ***SEF ratio*** | ***3-months*** | | ***12-months*** | | ***24-months*** | | ***36-months*** | |
| --- | --- | --- | --- | --- | --- | --- | --- | --- |
|  | ***TD50 (Gy)*** | ***m*** | ***TD50 (Gy)*** | ***m*** | ***TD50 (Gy)*** | ***m*** | ***TD50 (Gy)*** | ***m*** |
| <75% | 24.03 | 0.694 | 27.84 | 0.598 | 34.19 | 0.530 | 41.10 | 0.505 |
| <65% | 26.54 | 0.673 | 33.01 | 0.540 | 40.84 | 0.519 | 45.87 | 0.429 |
| <55% | 30.97 | 0.534 | 37.54 | 0.457 | 47.08 | 0.431 | 53.24 | 0.328 |
| <50% | 33.14 | 0.485 | 39.06 | 0.408 | 48.15 | 0.439 | 56.32 | 0.310 |
| **<45%** | **35.13** | **0.481** | **41.38** | **0.379** | **55.96** | **0.436** | **64.38** | **0.378** |
| <35% | 40.05 | 0.345 | 44.43 | 0.306 | 59.03 | 0.462 | 61.40 | 0.299 |
| <25% | 44.22 | 0.194 | 51.23 | 0.383 | 63.91 | 0.449 | 63.41 | 0.306 |

***Highlighted row (in bold) showing results using SEF ratio <45% that has previously been demonstrated to correlate best with salivary flow measurements***
